# Evaluation of Curcumin in *Candida albicans* Infection: Cytomorphometric Analysis, Antifungal Activity and Immunomodulatory Effects in *Galleria mellonella*

**DOI:** 10.3390/ph19060817

**Published:** 2026-05-23

**Authors:** Sukran Ozturk, Zehra Safi Oz

**Affiliations:** 1 Department of Pharmaceutical Microbiology, Faculty of Pharmacy, Zonguldak Bulent Ecevit University, 67600 Zonguldak, Türkiye; 2 Department of Medical Biology, Faculty of Medicine, Zonguldak Bulent Ecevit University, 67600 Zonguldak, Türkiye

**Keywords:** *C. albicans*, curcumin, cytomorphometry, *G. mellonella*, hemocyte, therapy, toxicity

## Abstract

**Background/Objectives:** The increasing prevalence of antifungal resistance among *Candida albicans* (*C. albicans*) strains necessitates the development of alternative therapeutic strategies. Curcumin (CUR), a natural polyphenolic compound, has attracted attention due to its antimicrobial and immunomodulatory properties. This study aimed to evaluate the antifungal activity of curcumin (CUR) and its effects on cellular and nuclear morphometric parameters (area, width, height, and perimeter), cytoplasmic area, and the nuclear-to-cytoplasmic ratio in an in vivo *Galleria mellonella* (*G. mellonella*) infection model. **Methods:** The experimental design consisted of four groups: (i) healthy control receiving phosphate-buffered saline (PBS), (ii) *C. albicans*-infected group (1.5 × 10^8^ CFU/mL), (iii) infected group treated with CUR (10 mg/kg), and (iv) healthy group treated with CUR. Survival was monitored for 96 h. Hemolymph samples were collected from larvae, smeared onto slides, and stained using May–Grünwald–Giemsa and Giemsa methods. Morphological evaluation and cytomorphometric analyses, including cellular area, nuclear area, cytoplasmic area, perimeter, width, height, and nucleus-to-cytoplasm ratio, were performed. **Results:** A higher dose (100 mg/kg) resulted in 100% mortality within 24 h and was therefore defined as lethal, whereas 10 mg/kg showed no toxicity in healthy larvae. Hemolymph was collected from surface-sterilized larvae, pooled per group, and a 10 µL aliquot was smeared onto slides. **Conclusions:** CUR exhibited significant antifungal activity against *C. albicans* and modulated host immune cell morphometry in the *G. mellonella* model. Its effects were dose-dependent, with potential cytotoxicity at higher concentrations. Further studies involving quantitative fungal burden analyses and mammalian models are required to clarify its therapeutic potential.

## 1. Introduction

Fungal infections have become a major global public health concern, particularly with the increasing number of immunocompromised individuals and the widespread use of broad-spectrum antimicrobial agents. Among opportunistic fungal pathogens, *C. albicans* is one of the leading causes of both invasive and mucosal infections worldwide. Its pathogenicity is closely associated with its ability to undergo morphological transitions between yeast, pseudohyphal, and hyphal forms, facilitating adhesion, tissue invasion, and immune evasion [1]. Additionally, structural components of the fungal cell wall, including β-glucans, chitin, and mannoproteins, play a crucial role in host–pathogen interactions and contribute to virulence [2,3].

Experimental infection models are essential tools for investigating host–pathogen interactions and evaluating the efficacy of novel antimicrobial agents [4]. Traditionally, mammalian models have been used; however, ethical concerns, high costs, and logistical constraints have led to the search for alternative in vivo systems. In recent years, *G. mellonella* larvae have emerged as a valuable and widely accepted model due to their functional and structural similarities to the mammalian innate immune system [5].

The *G. mellonella* immune response is characterized by both cellular and humoral components. The cellular response involves specialized cells known as hemocytes, which perform phagocytosis, nodulation, and encapsulation of pathogens, mirroring the activities of mammalian neutrophils and macrophages [6,7]. Simultaneously, the humoral response includes the production of antimicrobial peptides (AMPs), melanin through the phenoloxidase pathway, and reactive oxygen species (ROS) [8]. Notably, the ability of these larvae to be maintained at 37 °C allows the study of human-pathogenic fungi and bacteria under clinically relevant thermal conditions, making it an ideal bridge between in vitro assays and mammalian trials [9].

*C. albicans* is a polymorphic, yeast-like fungus that exists as a commensal organism within the human microbiota. However, it is also a formidable opportunistic pathogen, typically proliferating in individuals with a disrupted microbial balance or compromised immune competence [10]. In recent years, the global incidence of fungal infections caused by *Candida* species has risen significantly, accompanied by a concerning increase in resistance to conventional antifungal treatments [11].

Current therapeutic strategies for *C. albicans* infections rely on topical and systemic antifungal agents, primarily azoles, polyenes, and echinocandins. Nevertheless, the clinical utility of these drugs is often hampered by significant toxicity, adverse drug–drug interactions, and the rapid emergence of multidrug-resistant strains [12,13]. This alarming trend in antifungal resistance complicates treatment outcomes, particularly in immunocompromised patients, and underscores the urgent need to investigate novel and effective therapeutic alternatives [14]. Natural bioactive compounds, known for their diverse antimicrobial properties and lower toxicity profiles, have emerged as promising candidates for the development of alternative strategies to combat fungal infections [15].

Despite the availability of several antifungal agents, including azoles and echinocandins, the emergence of antifungal resistance among Candida species has become a serious clinical challenge [11]. Resistance to triazole antifungal drugs, in particular, has been widely reported and may significantly reduce treatment efficacy, especially in immunocompromised patients [12,16]. Furthermore, the toxicity, drug interactions, and limited efficacy of existing antifungal therapies highlight the urgent need for novel and safer therapeutic strategies [17].

Natural bioactive compounds have attracted increasing attention as potential antimicrobial agents due to their diverse pharmacological properties. Curcumin (CUR), a polyphenolic compound derived from the rhizomes of *Curcuma longa*, has been extensively studied for its antioxidant, anti-inflammatory, antimicrobial, and anticancer activities [18]. Several studies have demonstrated that curcumin exhibits antifungal activity against Candida species by disrupting membrane integrity, inhibiting virulence factors such as biofilm formation, and modulating intracellular signaling pathways [19,20,21]. In addition to its direct antifungal effects, curcumin has also been reported to modulate host immune responses, suggesting its potential role as a supportive therapeutic agent in infectious diseases [22].

Although the antifungal properties of curcumin have been widely investigated in vitro, its in vivo effects, particularly on host immune cells during fungal infection, remain insufficiently understood. One of the key limitations in current research is the lack of studies evaluating the cellular-level responses of host immune systems, especially morphological and morphometric changes, following curcumin treatment in infection models.

Cytomorphometry is a quantitative, computer-assisted technique that enables the evaluation of cellular characteristics such as size, morphology, and structural alterations. This method has been widely used to assess the effects of infectious agents and toxic compounds on various cell types [22,23]. However, despite the well-documented biological activities of curcumin, there is currently no comprehensive study investigating its cytomorphometric effects on hemocytes in the *G. mellonella* infection model.

Therefore, the present study aimed to evaluate the antifungal and immunomodulatory effects of curcumin in a *G. mellonella* model of *C. albicans* infection. Specifically, this study focused on analyzing morphological and cytomorphometric changes in hemocytes following curcumin treatment. By combining in vivo infection modeling with quantitative cellular analysis, this work seeks to provide novel insights into the dual role of curcumin as both an antifungal agent and a modulator of host immune responses.

## 2. Results

### 2.1. Determination of Minimum Lethal Concentration (MLC)

The toxicity of curcumin (CUR) was evaluated in both healthy and *C. albicans*-infected larvae at doses of 10 and 100 mg/kg. While the 100 mg/kg dose was tolerated by healthy larvae, it induced severe cellular deformation and hemocyte destruction in the infected group. Notably, 50% mortality was observed within 2 h of administration, reaching 100% mortality by 24 h. Consequently, 100 mg/kg was identified as the lethal threshold for infected larvae under these experimental conditions. In contrast, the 10 mg/kg dose demonstrated no larval mortality throughout the 96 h observation period. Cytological examination confirmed that 10 mg/kg CUR maintained hemocyte integrity while providing a therapeutic effect, thus establishing it as the optimal non-toxic concentration for subsequent assays [24].

### 2.2. In Vivo Activity of Curcumin

**Figure 1:**
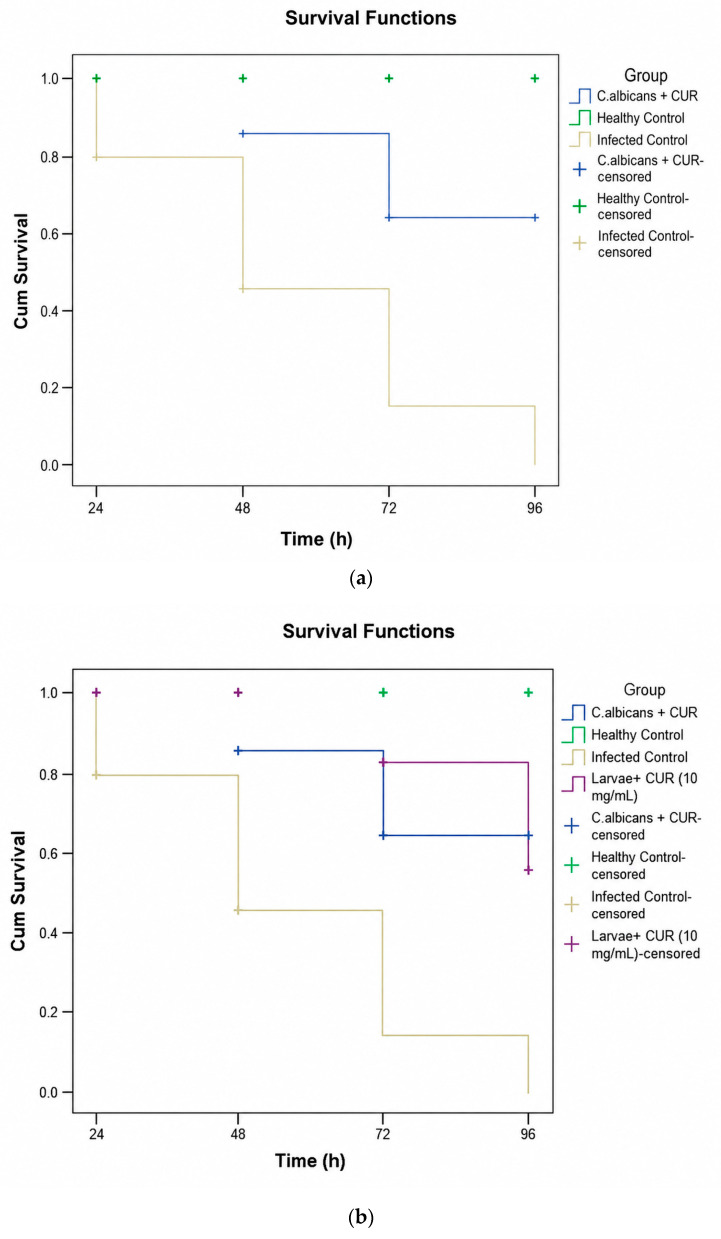

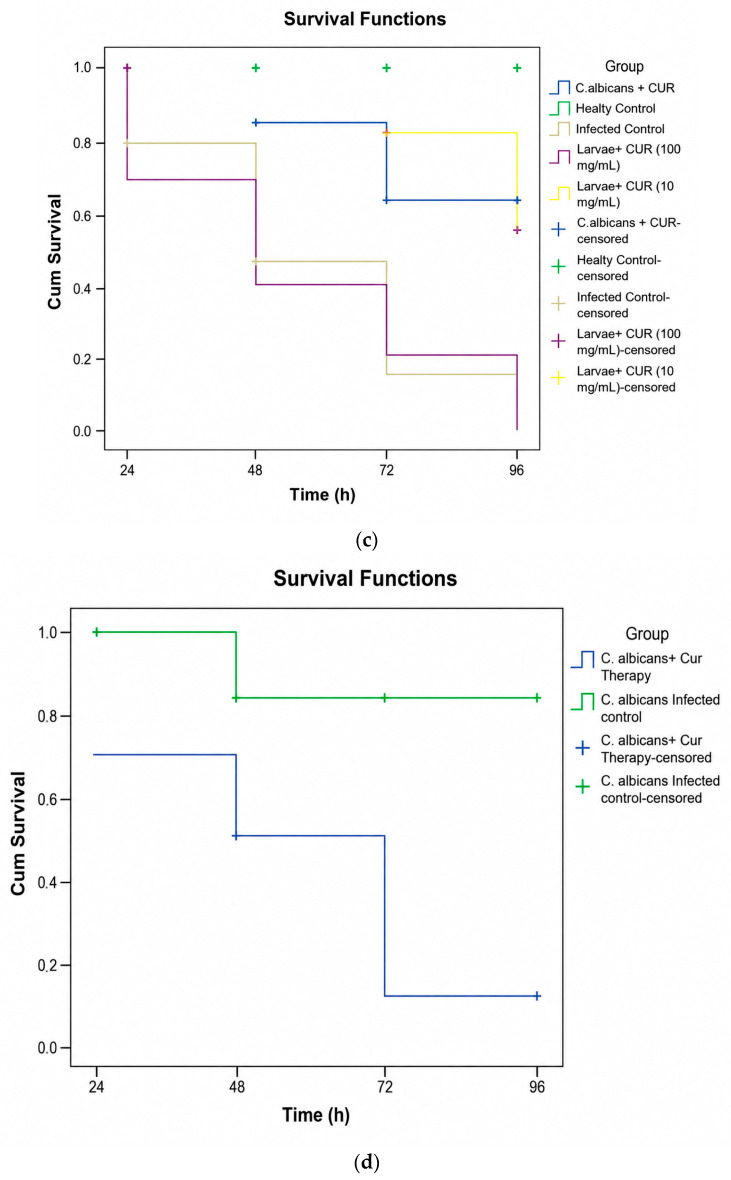
(**a**) Survival kinetics of *G. mellonella* larvae over a 96 h period following infection with *C. albicans*. Kaplan–Meier survival analysis revealed a significant dose-dependent protective effect of curcumin (CUR) treatment (*p* < 0.05). While the infection-only group (Group 2) exhibited a rapid decline in survival rates, reaching maximum mortality within the first 48–72 h, the groups treated with CUR demonstrated a prolonged lifespan. Specifically, the higher efficacy observed in CUR-treated larvae suggests that the compound not only exerts direct antifungal pressure on *C. albicans* but also potentially enhances the host’s innate immune resilience. The statistical significance (*p* < 0.05) confirms that the difference in survival outcomes between the untreated infected larvae and the CUR-treated groups is robust and not due to random variation, highlighting CUR as a potent immunomodulatory and antifungal candidate in this in vivo model. (**b**) Survival rates of *G. mellonella* larvae over 96 h. The graph compares the survival kinetics of larvae infected with *C. albicans* (1.5 × 10^8^ CFU/mL) with those receiving curcumin (CUR) treatment (10 mg/kg). The ‘healthy larvae + CUR’ group served as a toxicity control, demonstrating that CUR at 10 mg/kg did not adversely affect larval viability. A significant increase in survival was observed in infected larvae treated with CUR compared to the untreated infected group (*p* = 0.001), indicating the potent in vivo antifungal and protective efficacy of CUR against systemic Candidiasis. (**c**) Comparative survival analysis of *G. mellonella* under high-dose CUR administration. This panel illustrates the survival outcomes of larvae infected with *C. albicans* (1.5 × 10^8^ CFU/mL) and treated with CUR (10 mg/kg), alongside a high-dose safety group receiving CUR (100 mg/kg). The healthy larvae + CUR (100 mg/kg)’ group demonstrated that even at a tenfold increase over the therapeutic dose, CUR did not induce significant mortality or systemic toxicity in the host. The treatment group showed a statistically significant improvement in survival compared to the disease control (*p* = 0.001), further validating the therapeutic index and the safety profile of CUR in the *G. mellonella* model. (**d**) Comparative survival analysis of *G. mellonella* under appropriate CUR administration. This panel illustrates the survival outcomes of larvae infected with *C. albicans* (1.5 × 10^8^ CFU/mL) and treated with CUR (10 mg/kg). The treatment group demonstrated a statistically significant improvement in survival compared with the disease control group (*p* < 0.031).

*C. albicans*-infected larvae were evaluated 24 h after administration of the different treatment regimens, and statistically differences in mortality rates were observed between the infected group, the healthy group and the CUR therapy group (Figure 1a).

There was a statistically significant difference in survival rates among the groups, including the infected group (1.5 × 10^8^ CFU/mL *C. albicans*) and the CUR-treated groups (*p* < 0.05). Kaplan–Meier analysis was performed to estimate survival time, and subgroups were compared using the log-rank test (Figure 1a–c).

#### Survival of *Galleria mellonella* Larvae

Larval survival differed significantly among the experimental groups following *C. albicans* infection and curcumin treatment. All larvae in the healthy control group survived throughout the observation period. In contrast, the *C. albicans*-infected group showed a marked decrease in survival over time. Treatment with curcumin improved larval survival compared with the infected group, with a more pronounced protective effect observed at the lower dose. Kaplan–Meier survival analysis revealed statistically significant differences between the groups (log-rank test, *p* < 0.05). Appropriate dose CUR administration significantly improves the survival of *G. mellonella* larvae infected with *C. albicans* (1.5 × 10^8^ CFU/mL). Larvae treated with CUR (10 mg/kg) exhibited a statistically significant increase in survival compared to the disease control group (*p* < 0.031) (Figure 1d).

### 2.3. Morphological and Morphometric Analysis of G. mellonella Hemocytes

**Figure 2:**
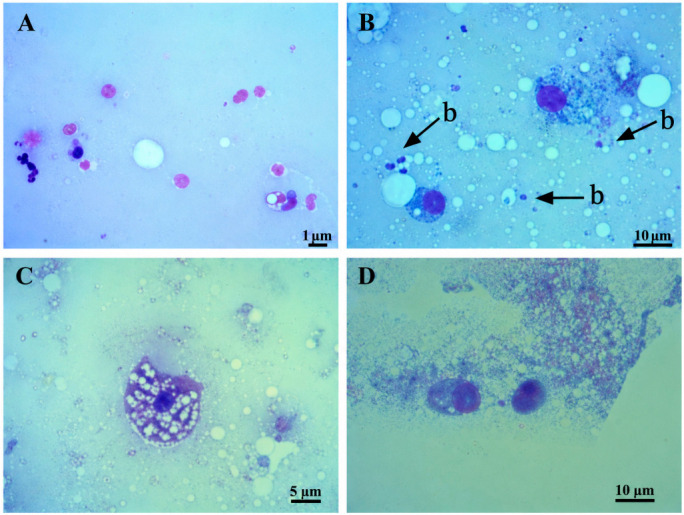
(**A**) Healthy *G. mellonella* hemocytes exhibiting normal cellular architecture, including intact cell membranes, clear cytoplasm, distinct cell borders, and centrally located nuclei in the control group. (**B**) *C. albicans* blastospores [b] and hemocytes observed in Group 2. (**C**) Hemocytes from the *C. albicans*-infected and CUR-treated group (Group 3). (**D**) Hemocytes and cellular debris in the CUR-treated group [MGG staining].

Light microscopic examination of hemocyte smears revealed distinct morphological alterations among the groups (Figure 2). All cytological smears were evaluated for the presence of hemocytes and *C. albicans* structures (blastospores and hyphae). In the healthy control group, hemocytes exhibited typical morphological features and normal cellular architecture, characterized by well-defined boundaries, intact cell membranes, clear and well-defined cytoplasm, and centrally located nuclei (Figure 2A). In the *C. albicans*-infected group, blastospores were clearly identified, often surrounded by a distinct halo or localized near hemocytes, indicating active infection. In contrast, hemocytes obtained from *C. albicans*-infected larvae showed pronounced morphological changes, including cytoplasmic shrinkage, nuclear condensation, membrane irregularities, and increased cellular debris (Figure 2B). Notably, in the group infected with *C. albicans* and treated with CUR (10 mg/kg), comprehensive examination of the smears revealed an absence of visible fungal blastospores or hyphae. Curcumin-treated larvae displayed notable alterations in hemocyte morphology compared with the infected group. (Figure 2C), suggesting that CUR treatment effectively limited fungal proliferation in vivo [25]. At both doses, CUR treatment resulted in reduced cellular deformation and partial restoration of hemocyte structure. However, in the high-dose CUR group (100 mg/kg), significant cellular debris and hemocyte fragmentation were observed, confirming localized toxic effects (Figure 2D) [24]. At the higher dose, some parameters indicated increased cellular damage, consistent with the toxic effects observed microscopically.

Comparison of nuclear and cellular morphometric parameters among experimental groups is given in Table 1. In the *C. albicans*-infected group, nuclear and cellular parameters (area, width, height, and perimeter), as well as cytoplasmic area, were significantly higher than those in the control group.In contrast, the nucleus-to-cytoplasm (N:C) ratio differed significantly between the healthy control and infected groups, indicating that it may serve as a sensitive marker of infection-induced stress (*p* < 0.05). In the CUR-only (10 mg/kg) group, nuclear and cellular parameters, including the N:C ratio and cytoplasmic area, were significantly lower than those of the healthy control (*p* < 0.05).

In the *C. albicans*-infected + CUR-treated group, a significant reduction in nuclear parameters and the N:C ratio was observed compared to the control (*p* < 0.05), while the cytoplasmic area showed an increase (*p* < 0.05). Statistically significant differences were confirmed between the following groups: Infected vs. CUR-treated: Significant difference in hemocyte morphology (*p* < 0.05). CUR-only vs. Infected + CUR-treated: Statistically significant variation in cellular dimensions (*p* = 0.001). Infected + CUR-treated vs. healthy control: Highly significant morphological shift (*p* < 0.05), indicating that while CUR mitigates infection, it also modulates the baseline hemocyte state [22,26].

### 2.4. Assessment of Cellular Damage Patterns

**Figure 3:**
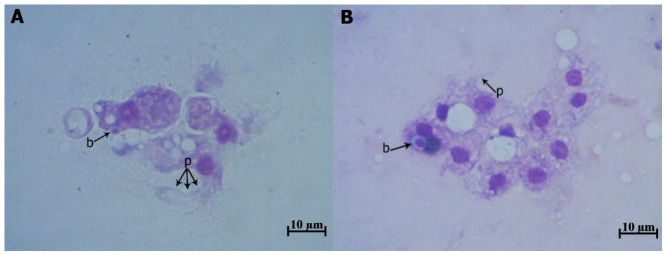

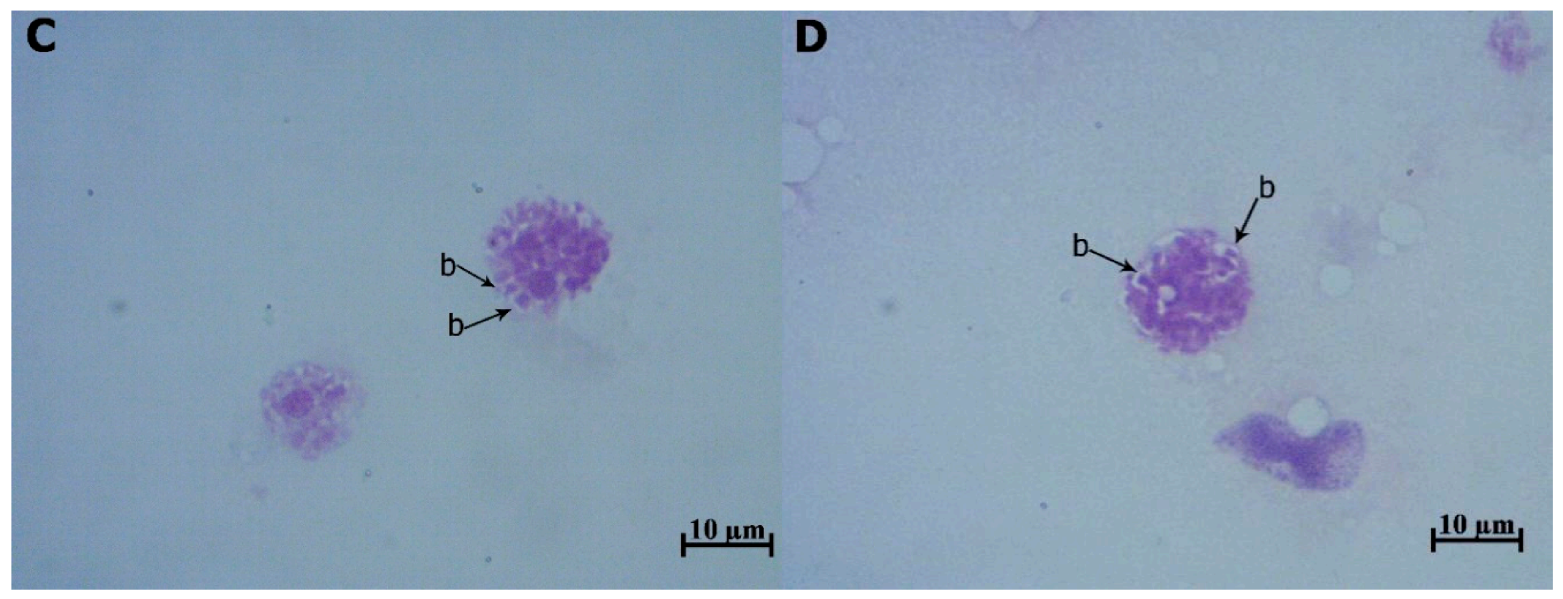
In *C. albicans*-infected and CUR-treated *G. mellonelle* (Group 3) (**A**,**B**). *C. albicans* blastospores (b) and hemocytes with pseudopodia (p). (**C**,**D**) *C. albicans* blastospores (b) phagocytosed by *G. mellonella* hemocytes [MGG staining].

Based on combined morphological and cytomorphometric findings, hemocytes from *C. albicans*-infected larvae predominantly exhibited changes suggestive of cellular stress and damage. Curcumin treatment was associated with morphological patterns indicative of both apoptotic-like and necrotic-like changes, depending on the dose. In addition to these morphological changes, light microscopic observations revealed that curcumin treatment enhanced the phagocytic capacity of hemocytes in *C. albicans*-infected *G. mellonella* larvae. This suggests that while curcumin induces dose-dependent structural changes, it simultaneously exerts an immunomodulatory effect by promoting the pathogen-engulfing efficiency of the immune cells, indicating an enhancement of the cellular immune response despite the observed morphological shifts (Figure 3).

## 3. Discussion

Antimicrobial resistance has emerged as a major global health concern, particularly in fungal infections where therapeutic options are increasingly limited. The rise in antifungal resistance among *C. albicans* strains, especially against azole compounds, significantly compromises treatment efficacy and necessitates the development of alternative or adjunctive therapeutic strategies [16,27]. In this context, natural compounds such as CUR have gained attention due to their diverse biological activities, including antimicrobial and immunomodulatory effects [28].

The present study investigated the antifungal and immunomodulatory effects of curcumin using an in vivo *G. mellonella* infection model, with a particular focus on cytomorphometric changes in hemocytes. Although the antifungal activity of curcumin has been widely demonstrated in in vitro studies, its effects on host immune cells under in vivo infection conditions remain insufficiently characterized. Therefore, this study contributes to the literature by combining survival analysis with detailed morphological and morphometric evaluation of hemocytes.

The *G. mellonella* model is increasingly used as an alternative in vivo system due to its functional similarities to the mammalian innate immune system, particularly in terms of cellular immune responses mediated by hemocytes [7,22]. Hemocytes play a central role in phagocytosis, encapsulation, and pathogen elimination, making them suitable for evaluating host immune responses [6]. However, it is important to note that this model does not fully replicate mammalian pharmacokinetics, including drug absorption, metabolism, and systemic distribution. Curcumin, in particular, is known to exhibit poor bioavailability and rapid metabolism in humans, which are not reflected in the Galleria model [17,28]. Therefore, extrapolation of these findings to human systems should be approached with caution.

In the present study, curcumin exhibited a clear dose-dependent effect. The high dose (100 mg/kg) resulted in rapid larval mortality and pronounced cellular damage, indicating significant toxicity. In contrast, the lower dose (10 mg/kg) did not adversely affect larval survival and was therefore selected as the therapeutic dose. These findings are consistent with previous studies demonstrating that curcumin may induce cytotoxic effects at higher concentrations while exerting beneficial biological effects at lower doses [29]. Furthermore, previous reports indicate that the effective antifungal concentration of curcumin ranges between 64 and 256 mg/L [30]. Although direct comparison between in vitro and in vivo concentrations is challenging, the selected dose in this study appears to fall within a biologically relevant range. Appropriate curcumin (CUR; 10 mg/kg) administration significantly improved the survival of *G. mellonella* larvae infected with *C. albicans* (1.5 × 10^8^ CFU/mL), as demonstrated in Figure 1d (*p* < 0.031). This finding suggests that CUR exerts a protective effect even under conditions of high fungal burden. The observed survival benefit may be attributed to the dual role of curcumin, including its reported antifungal properties and its capacity to modulate host immune responses. In the *G. mellonella* model, enhanced survival is often associated with reduced pathogen load and/or attenuation of excessive immune activation, such as melanization. Therefore, CUR treatment may contribute to improved host resilience by balancing antifungal activity with immunomodulatory effects. These results are consistent with previous studies highlighting the therapeutic potential of curcumin against fungal infections and support its further investigation as an adjunctive antifungal agent.

Survival analysis revealed that curcumin treatment significantly improved the survival of *C. albicans*-infected larvae compared with untreated infected controls. These findings suggest a protective role of curcumin during fungal infection. Importantly, preliminary experiments demonstrated that the selected low dose of curcumin did not affect the survival of healthy larvae, supporting the notion that the observed protective effect is associated with its interaction with infection rather than direct toxicity.

A notable observation in this study was the absence of detectable blastospores and hyphal structures in hemolymph samples obtained from curcumin-treated larvae. This finding supports the antifungal activity of curcumin and is consistent with previous studies demonstrating its inhibitory effects on fungal growth and virulence factors [20]. CUR has been reported to disrupt fungal cell membrane integrity, interfere with intracellular signaling pathways, and modulate oxidative stress responses [19]. These mechanisms may contribute to the reduced presence of fungal elements observed in the treated groups.

However, it is important to acknowledge that fungal burden was not quantitatively assessed using colony-forming unit (CFU) analysis. While morphological observations strongly suggest a reduction in fungal load, the absence of quantitative data represents a limitation of the study. Future studies incorporating CFU-based assays will be essential to validate these findings and provide a more comprehensive evaluation of antifungal efficacy.

Cytomorphometric analysis revealed significant alterations in hemocyte parameters following infection and curcumin treatment. Infected larvae exhibited increased cellular and nuclear dimensions, which may reflect cellular activation in response to infection. These findings are consistent with previous studies indicating that immune cell activation is associated with morphological changes during host–pathogen interactions [23]. In contrast, curcumin treatment resulted in decreased nuclear and cellular dimensions, as well as a reduced nucleus-to-cytoplasm ratio. These changes are indicative of apoptotic-like cellular responses, including cellular shrinkage and nuclear condensation.

In addition to apoptotic-like changes, the presence of lysed hemocytes and cellular debris suggests that necrotic-like cell death may also occur, particularly at higher concentrations of curcumin. These findings are consistent with previous reports demonstrating that curcumin can induce both apoptosis and necrosis depending on concentration and cellular context [31]. One proposed mechanism involves the generation of reactive oxygen species (ROS), leading to mitochondrial dysfunction, cytochrome c release, and activation of apoptosis-related pathways [32]. At higher concentrations, excessive oxidative stress may overwhelm cellular defense mechanisms, resulting in necrotic cell death [29].

The present study also suggested a potential increase in phagocytic activity based on morphological observations, such as the apparent interaction between hemocytes and fungal elements. However, it should be emphasized that no direct functional assays were performed to quantify phagocytosis. Therefore, this interpretation remains speculative and requires further validation using dedicated phagocytic assays.

Another important finding of this study is the dual effect of curcumin. While it demonstrated antifungal activity and improved survival in infected larvae, it also induced cytotoxic effects on host hemocytes. This highlights the complexity of curcumin’s biological effects and underscores the importance of dose optimization. Similar dual effects have been reported in previous studies, where curcumin exhibited both protective and cytotoxic properties depending on concentration [33].

Overall, the results of this study demonstrate that curcumin exhibits antifungal activity against *C. albicans* and modulates host immune responses in the *G. mellonella* model. However, its effects are dose-dependent and involve both beneficial and cytotoxic mechanisms. Further studies incorporating molecular analyses, quantitative fungal burden measurements, and mammalian models are required to better understand the therapeutic potential and safety profile of CUR.

## 4. Materials and Methods

### 4.1. Candida albicans Strains

Fluconazole-resistant *C. albicans* strains used in this study were obtained from the clinical microbiology culture collection of Zonguldak Bülent Ecevit University Hospital. The identification of the isolates was confirmed using standard mycological procedures. Fluconazole resistance was determined by the broth microdilution method to establish the Minimum Inhibitory Concentration (MIC), following the guidelines established by the Clinical and Laboratory Standards Institute (CLSI) M27-A4 document [34]. According to these criteria, isolates with an MIC value of ≥64 μg/mL were categorized as resistant [35]. The strains were maintained and subcultured in the culture collection of the Department of Pharmaceutical Microbiology, Faculty of Pharmacy, Zonguldak Bülent Ecevit University.

### 4.2. Preparation of Curcumin Solution

Curcumin (CUR) was obtained from a commercial supplier. A stock solution was prepared by dissolving curcumin in dimethyl sulfoxide (DMSO) due to its low aqueous solubility. The stock was subsequently diluted in sterile phosphate-buffered saline (PBS) to obtain the desired working concentrations [28].

The final concentration of DMSO in all injections was kept below 1% (*v*/*v*) to avoid solvent-related toxicity [36]. Phosphate-buffered saline (PBS), a physiologically compatible and non-toxic solution commonly used as a vehicle in *G. mellonella* injection models, was administered to establish the healthy control group.

### 4.3. Galleria mellonella Larvae

**Figure 4:**
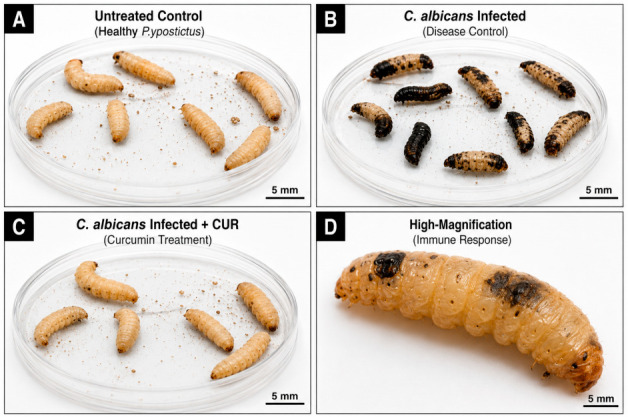
Experimental evaluation of *Galleria mellonella* infection and treatment response. (**A**) Untreated control larvae showing normal morphology and pigmentation. (**B**) Larvae infected with *Candida albicans* exhibiting pronounced melanization associated with infection. (**C**) Representative images of C. albicans-infected larvae treated with curcumin (CUR), showing partial reduction of melanization compared with the disease control group. (**D**) High-magnification image of an infected larva demonstrating localized melanization associated with the innate immune response. Scale bars are shown in each panel.

*G. mellonella* larvae were obtained from the Pharmaceutical Microbiology Laboratory. Final instar larvae weighing 0.2–0.3 g, without melanization or visible damage, were selected for the experiments. Larvae were maintained on a diet of honey, honeycomb, and bran under controlled laboratory conditions. *G. mellonella* larvae treated with curcumin are shown in Figure 4.

### 4.4. Determination of Minimum Lethal Concentration of Curcumin (MLC)

To determine the minimum lethal concentration (MLC) of curcumin, larvae were randomly divided into groups (*n* = 10 per group), and each experiment was performed in triplicate [24,37].

Based on preliminary experiments and literature data, two concentrations (10 mg/kg and 100 mg/kg) were selected:

- 100 mg/kg to evaluate toxicity threshold,
- 10 mg/kg as a potential therapeutic dose.

Prior to injection, the larval proleg was disinfected with 70% ethanol. A total of 10 µL of curcumin solution was injected into the hemocoel via the last left proleg using a sterile 1 mL insulin syringe [38].

Although Hamilton syringes are commonly used, insulin syringes were preferred due to:

- ease of handling;
- availability;
- the ability to deliver precise small volumes.

Larvae were incubated at 28 °C, and survival was monitored at 24, 48, 72, and 96 h.

### 4.5. Infection and Survival Assay

A standardized inoculum of *C. albicans* (1.5 × 10^8^ CFU/mL) was prepared in sterile PBS. Each larva was injected with 10 µL of the suspension into the hemocoel (31).

Two hours post-infection, treatment was administered.

Experimental groups (n = 10 per group; experiments repeated three times):

- Group 1: Healthy larvae (PBS);
- Group 2: *C. albicans*-infected larvae;
- Group 3: *C. albicans*-infected + CUR (10 mg/kg)-treated larvae;
- Group 4: Healthy larvae+ CUR (10 mg/kg)-treated.

Larvae were incubated at 37 °C, and survival rates were recorded every 24 h for 96 h, in accordance with standardized *G. mellonella* infection protocols [39].

### 4.6. Hemolymph Collection and Staining Procedures

Prior to hemolymph extraction, larvae were surface-sterilized with 70% (*v*/*v*) ethanol to minimize microbial contamination. Hemolymph was collected by puncturing the last proleg of non-melanized larvae with a sterile needle. To prevent spontaneous melanization and coagulation, the collected fluid was immediately transferred into chilled microcentrifuge tubes [26]. To account for biological variation and ensure representative sampling, hemolymph from ten larvae per group was pooled. A 10 µL aliquot of the pooled hemolymph was spread onto glass slides in a single direction to create a uniform thin smear. The slides were air-dried and subsequently processed for May–Grünwald–Giemsa (MGG) and Giemsa staining to evaluate hemocyte morphology and fungal load [23]. MGG staining, which works on the same principle as Romanowsky stains, facilitates detailed visualization of cellular morphology and assesses the cytoplasm, granules, and vacuoles in detail. Giemsa staining enhances nuclear and cytoplasmic contrast, allowing for clearer identification of cellular components and potential cellular changes. These staining methods were used together to provide a more comprehensive and reliable assessment of *G. mellonella* hemocytes, including morphological evaluation, morphometric analysis, and characterization of the immune response [7,40].

#### 4.6.1. May–Grünwald–Giemsa Staining

Air-dried slides were stained with May–Grünwald solution for 2.5 h, followed by Giemsa solution (1:4 dilution in distilled water) for 10 min. After rinsing with distilled water, slides were dehydrated in xylene and mounted using Entellan for permanent storage [7].

#### 4.6.2. Giemsa Staining

Air-dried slides were fixed in methanol for 5 min and stained with Giemsa solution for 35 min, as previously described, allowing clear visualization of hemocyte nuclei and cytoplasmic granules [40].

### 4.7. Morphologic Analysis

All slides were examined under ×40 magnification for general morphological assessment and under ×100 magnification for the presence of hemocytes and *C. albicans* blastospore/hyphe (in *C. albicans*-infected slides). High-resolution images were captured using a digital camera (Axio Cam ERc5s, Carl Zeiss, Oberkochen, Germany). All stained slides were photographed, and the images were digitally stored for analysis.

### 4.8. Cytomorphometric Analysis

**Figure 5:**
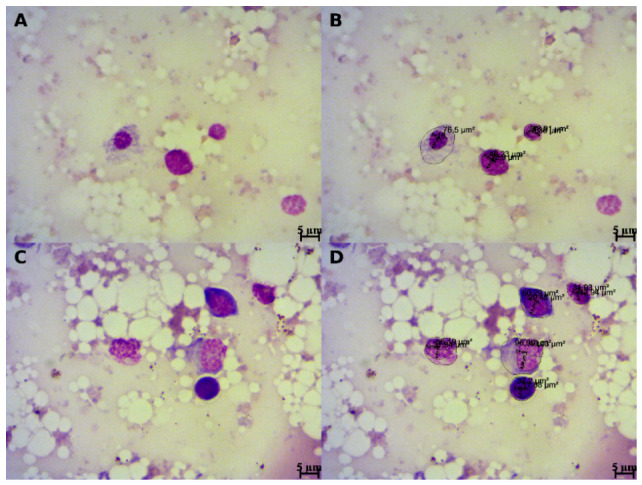
(**A**,**C**) *G. mellonella* hemocyte smear images in Group 1 (**B**,**D**) Morphometric measurements of *G. mellonella* hemocytes in Group 1.

Morphometric measurements of *G. mellonella* hemocytes were performed on photographed areas from all groups using AxioVision LE Rel. 4.8 software and an Axio Lab.A1 microscope (Carl Zeiss, Oberkochen, Germany). Fifty clearly defined hemocytes were measured using an eyepiece micrometer for cytomorphometric evaluation in each group (Figure 5). Cell boundaries and nuclear perimeters were manually outlined using a digital cursor. The cell area (CA) and nuclear area (NA) were measured directly. The cytoplasmic area (CyA) and nucleus-to-cytoplasm (N:C) ratio, which serve as indicators of cellular activation and metabolic state, were calculated using the following equations [22]:

CyA = CA − NA

Ratio = \frac{NA}{CyA}

### 4.9. Statistical Analysis

Larval survival data were analyzed using the Kaplan–Meier method, and differences between groups were determined using the log-rank (Mantel–Cox) test [39]. For cytomorphometric data, normality was assessed using the Shapiro–Wilk test. Since the data exhibited a non-parametric distribution, variables were expressed as median (min–max). Statistical significance among multiple groups was analyzed using the Kruskal–Wallis test followed by Dunn’s post hoc test for multiple comparisons. All analyses were performed using IBM SPSS Statistics (version 19.0), and *p* < 0.05 was considered statistically significant.

## 5. Conclusions

The present study demonstrated that curcumin exhibits antifungal activity against *C. albicans* and modulates host immune responses in the *G. mellonella* larval infection model. Treatment with curcumin resulted in a significant improvement in larval survival and in the absence of detectable fungal elements in hemolymph, supporting its antifungal potential.

In addition, cytomorphometric and morphological analyses revealed that curcumin induces significant alterations in hemocyte structure, including reductions in nuclear and cellular dimensions and changes consistent with apoptotic-like responses. However, the presence of lysed cells and cellular debris, particularly at higher doses, indicates that curcumin may also exert cytotoxic effects depending on concentration.

These findings highlight the dual role of curcumin as both an antifungal and a biologically active compound capable of influencing host immune cells. While the observed protective effects are promising, the dose-dependent cytotoxicity underscores the importance of careful dose optimization.

It is important to note that the antifungal effect observed in this study was based on morphological evaluation, and quantitative fungal burden analysis was not performed. Therefore, future studies should incorporate colony-forming unit (CFU) assays, molecular analyses, and mammalian models to further elucidate the mechanisms of action and evaluate the clinical relevance of curcumin.

Overall, CUR represents a promising candidate as a supportive therapeutic agent against *Candida* infections; however, its complex biological effects and pharmacokinetic limitations warrant further investigation.

## Figures and Tables

**Table 1:** Comparison of nuclear and cellular morphometric parameters among experimental groups (median [min–max], n = 10 per group).

Parameters	Group 1 (n = 10)	Group 2 (n = 10)	Group 3 (n = 10)	Group 4 (n = 10)	*p* Value
Nuclear area (µm^2^)	40.14 [10.21–78.30]	41.83 [12.20–87.87]	14.73 [4.40–78.30]	4.70 [1.18–63.87]	<0.05
Nuclear width (µm)	7.54 [3.86–13.65]	7.56 [4.09–11.23]	4.30 [2.61–10.00]	2.46 [1.23–8.59]	<0.05
Nuclear height (µm)	6.87 [3.68–11.48]	7.00 [3.83–11.05]	4.47 [1.93–10.17]	2.37 [1.22–11.23]	<0.05
Nuclear perimeter (µm)	23.43 [11.82–36.28]	23.65 [12.70–34.83]	14.38 [7.96–32.78]	8.20 [4.15–29.23]	<0.05
Cellular area (µm^2^)	84.74 [34.50–311.70]	102.90 [38.76–231.81]	71.00 [20.75–204.00]	25.38 [3.80–14,735]	<0.05
Cellular width (µm)	11.04 [6.14–24.60]	12.04 [6.96–18.07]	9.73 [4.78–24.56]	5.79 [1.75–19.47]	<0.05
Cellular height (µm)	10.48 [5.79–17.65]	11.21 [6.09–20.17]	8.86 [4.74–15.13]	5.58 [2.28–11.93]	<0.05
Cellular perimeter (µm)	35.35 [22.88–65.78]	37.18 [22.57–59.17]	31.14 [16.60–59.74]	19.36 [8.04–49.31]	<0.05
Cytoplasmic area (µm^2^)	43.02 [9.61–246.49]	66.68 [21.27–167.96]	51.16 [13.76–191.17]	20.19 [−39.01–83.49]	<0.05
Nuclear area/cytoplasmic area	0.78 [0.18–5.46]	0.56 [0.17–1.28]	0.29 [0.07–1.70]	0.22 [−1.15–1.62]	<0.05

Group 1: Healthy control larvae + PBS; Group 2: *C. albicans*-infected larvae; Group 3: *C. albicans*-infected larvae treated with CUR; Group 4: Healthy larvae treated with CUR. Data are presented as median (min–max). Statistical analysis was performed using the Kruskal–Wallis test followed by Dunn’s post hoc test (*p* < 0.05).

## Data Availability

The original contributions presented in this study are included in the article. Further inquiries can be directed to the corresponding author.
